# Analytical Expressions for Ising Models on High Dimensional Lattices

**DOI:** 10.3390/e23121665

**Published:** 2021-12-10

**Authors:** Boris Kryzhanovsky, Leonid Litinskii, Vladislav Egorov

**Affiliations:** Center of Optical Neural Technologies, Scientific Research Institute for System Analysis RAS, Nakhimov Ave, 36-1, 117218 Moscow, Russia; kryzhanov@mail.ru (B.K.); rvladegorov@rambler.ru (V.E.)

**Keywords:** Ising model on hypercube, free energy, density of states, *m*-vicinity method

## Abstract

We use an *m*-vicinity method to examine Ising models on hypercube lattices of high dimensions d≥3. This method is applicable for both short-range and long-range interactions. We introduce a small parameter, which determines whether the method can be used when calculating the free energy. When we account for interaction with the nearest neighbors only, the value of this parameter depends on the dimension of the lattice d. We obtain an expression for the critical temperature in terms of the interaction constants that is in a good agreement with the results of computer simulations. For d=5,6,7, our theoretical estimates match the numerical results both qualitatively and quantitatively. For d=3,4, our method is sufficiently accurate for the calculation of the critical temperatures; however, it predicts a finite jump of the heat capacity at the critical point. In the case of the three-dimensional lattice (d=3), this contradicts the commonly accepted ideas of the type of the singularity at the critical point. For the four-dimensional lattice (d=4), the character of the singularity is under current discussion. For the dimensions d=1, 2 the *m*-vicinity method is not applicable.

## 1. Introduction

Statistical physics provides effective methods of analysis, allowing us to investigate large systems of elementary “agents” and to determine macroscopic characteristics—in particular, the free energy based on interactions between the “agents”. If we know the free energy—by means of computer simulations or theoretical calculations—we can calculate such properties of the system as the internal energy, magnetization, heat capacity, and susceptibility. The singularities of the temperature dependences of these characteristics define the critical temperatures at which the internal restructurings take place in the system and phase transitions occur.

Such results (even if they are not quite accurate) are important, and not only for physicists. In the second half of the 1980s, the statistical physics methods were applied to estimate the storage capacity of the Hopfield neural network. Later on, a lot of investigations in the field of neural science based on the statistical physics followed (see, for example, [1,2,3]). At the same time, the statistical physics methods became popular in the combinatorial optimization problems [4,5,6]. Starting from the mid-1990s, a new scientific branch named econophysics appeared. In econophysics, the statistical physics methods are the main instruments for analyzing economic models [7,8].

Physics provides a wide variety of methods for the calculation of the free energy, from computer simulations (where one uses the Metropolis or the Wang-Landau algorithms [9,10,11]) to cumbersome theoretical approaches of the type of the renormalization group or the transfer-matrix methods [12,13,14].

In the present paper, we sum up the results obtained when developing the *m*-vicinity method (at first, we called it “the *n*-vicinity method”, but then it became clear that the more appropriate term was “the *m*-vicinity method”) for analysis of the Ising systems. Our method allows us to calculate the free energy for an arbitrary connection matrix. We present a review of our results for the Ising models on hypercubic lattice of the dimensions d=3,4,5,6, and 7. The dimensions d=1 and d=2 are absent in this list because this method is not applicable for such lattices.

There is an enormous number of papers of theoretical or numerical studies on the behavior of spin systems on specific types of lattices. Here, we cite only the papers that we used in the course of our work. Cubic lattices are studied, for example, in [9,15,16,17,18,19]. Four-dimensional lattices are discussed in [20,21,22]. Studies on higher-dimensional systems are very rare. Our single source on d≥5 was [23].

In the next section, we justify the main approximation of our method. It consists of the substitution of the Gaussian distribution in place of the unknown state distribution of the given Hamiltonian.

In Section 3, we show that the Gaussian approximation is the first-order term in the expansion of the density of states in a perturbation theory series in a small parameter $\varepsilon_{\max}$. In the case of the planar lattice (*d* = 2), $\varepsilon_{\max}\approx 0.7$, and this value is not sufficiently small. When the lattice dimension d increases, the value of $\varepsilon_{\max}$ decreases quickly, and the Gaussian approximation works very well.

In Section 4, we present in detail our results obtained with the aid of Gaussian approximation of the density of states. The mean and the variance of the Gaussian distribution that we use coincide with the first and the second moments of the density of states of the given system. We define the boundaries of the method applicability and obtain analytical expressions for the critical characteristics of the system. In particular, they are the critical value of the inverse temperature and the jump of the heat capacity. Our analytical results match quite accurately with the results of computer simulations. We find out that the higher the dimension of the lattice, the better our estimates; when d≥5, the relative error is of the order of the tenth or the hundredth percent.

In Section 5, we check whether the account of the second-order terms of the perturbation theory improves our results for the critical temperature. Here, we approximate the density of states with a distribution whose first three moments coincide with the moments of the state distribution of the system. (To do this a more advanced knowledge of the mathematical statistics is necessary [24,25].) We find that, while the role of second order parameters is negligible, we can achieve almost perfect agreement with computer simulations by introducing an adjustable parameter.

In Section 6, we present a detailed comparison of the theoretical results and computer simulations for the Ising model on the cubic lattice (d=3). For such a lattice, we examine the role of the long-range interaction; in particular, we discuss the interactions with the next-nearest neighbors and the next-next-nearest neighbors.

Finally, in Section 7, we sum up the strengths and weaknesses of the *m*-vicinity method. The details of the calculations are in the Appendix A.

## 2. Main Approximation of *m*-Vicinity Method

Let us examine the Ising model on a multidimensional cubic lattice, which is a system of N spins $s_{i}=\left\{\pm 1\right\}$, i=1,2,…N situated at the nods of a hypercubic lattice. In what follows, we assume the periodic boundary conditions.

The Hamiltonian of the system is

$${Ε}_{H}=E-mH,\;E=-\frac{1}{2N}\sum_{i,j=1}^{N}J_{ij}s_{i}s_{j},\;m=\frac{1}{N}\sum_{i=1}^{N}s_{i},$$

where $J={\left(J_{ij}\right)}_{1}^{N}$ is a connection matrix, *H* is a magnetic field, and *m* is a magnetization of the state $s=(s_{1},s_{2},\ldots ,s_{N})$. The partition function of the system is

$$Z=\sum_{E}D(E)\exp\left(-N\beta E_{H}\right),$$

where β is the inverse temperature, the summation is carried out over all the values of the energy E, and D(E) is the density of states (the degeneracy of the energy states).

In the general case, we do not know the energy distribution D(E). It seems that we can define it with the aid of the central limit theorem. Indeed, the value of *E* is the sum of N(N−1)/2 weakly connected random variables, and the Gaussian distribution

(1) $$2^{N}\sqrt{\frac{N}{2\pi \sigma_{0}}}\exp\left(-\frac{1}{2}N\frac{E^{2}}{\sigma_{0}^{2}}\right),\;\mathrm{where}\;\sigma_{0}^{2}=\frac{1}{2N}\sum_{i,j=1}^{N}J_{ij}^{2},$$

describes correctly the central part of the distribution D(E). However, Equation (1) is not applicable at the tails of the true distribution, while the tails provide the main contribution to the formation of the phase transition. Many authors mentioned this fact (see [15]). The *m*-vicinity method allows us to overcome this difficulty. The essence of the method is as follows. We divide the set of $2^{N}$ configurations into N+1 subsets $\Omega_{m}$, which will be called the *m*-vicinities. The *m*-vicinity $\Omega_{m}$ contains all the configurations s with the same magnetization m. These configurations s differ from the configuration of the ground state $s_{0}=(1,1,\ldots ,1)$ by opposite signs of n spins, where n=N(1−m)/2 and the number of such configurations is equal to $\left(\begin{array}{c} N\\ n \end{array}\right)$.

The density D(E,m) is the energy distribution for a given *m*-vicinity, and the partition function of the system is

(2) $$Z=\overset{N}{\underset{n=0}{\sum }}\underset{E}{\sum }D(E,m)\exp[-N\beta (E-mH)].$$

In the general case, we do not know the true distribution D(E,m). However, we do know the exact values of its mean energy $E_{m}$ and its variance, which we denote as $N^{-1}\sigma_{m}^{2}$ (see Appendix A or [26,27,28]). In the case of the Ising model on the hypercube, these expressions in the limit N→∞ are sufficiently simple,

(3) $$E_{m}=E_{0}m^{2},\;N^{-1}\sigma_{m}^{2}=N^{-1}\sigma_{0}^{2}\cdot {\left(1-m^{2}\right)}^{2},\;E_{0}=-\frac{1}{2N}\sum_{i,j=1}^{N}J_{ij},$$

where $E_{0}=E(s_{0})$ is the energy of the ground state $s_{0}$ of the Ising Hamiltonian.

Let us explain the purpose of dividing the whole set of configurations into *m*-vicinities. In the vicinity $\Omega_{m}$, the energy E behaves as a random value and due to the central limit theorem, we can approximate accurately the central part of D(E,m) by Gaussian distribution with the mean $E_{m}$ and the variance $N^{-1}\sigma_{m}^{2}$:

(4) $$D(E,m)=\frac{\sqrt{N}}{\sqrt{2\pi }\sigma_{m}}\;\left(\begin{array}{l} N\\ n \end{array}\right)\;\exp\left[-\frac{1}{2}N{\left(\frac{E-E_{m}}{\sigma_{m}}\right)}^{2}\right],\;n=N\left(1-m\right)/2.$$

It is evident that the sum $\sum_{m}D(E,m)$ differs from the Gaussian distribution (1) and it better describes the tails of the true distribution.

Let us note here that in the limit $\sigma_{m}\to 0$, we can replace the exponent in Equation (4) by a delta function $\delta (E-E_{m})$. Then Equation (2) takes the classical form known from the mean field theory, which provides the Bragg–Williams results [29]. However, the value of $\sigma_{m}$ differs from zero for all the types of the connection matrices (an exclusion is the case of the complete graph when all the matrix elements are equal—see Equation (A4)) and the replacement of the Gaussian (4) by the delta function is not correct. It is the account for the value $\sigma_{m}\neq 0$ that leads to a much better agreement of theoretical estimates and results of simulations. Of course, the distribution D(E,m) is not purely Gaussian since its higher odd moments are not equal to zero (see Appendix A); however, their contribution is sufficiently small. In what follows, we analyze when the Gaussian approximation (4) is applicable and how a deviation of the density of states D(E,m) from Equation (4) influences the results. In the next section, we show that expression (4) is the first-order term of the perturbation theory in a small parameter ${\left(2q\right)}^{-1/2}$, where q is an effective number of neighbors (see Equation (16)).

## 3. Small Parameter in *m*-Vicinity Method

The basis of the *m*-vicinity method is the abovementioned approximate description of the density of states. To analyze the approximation, we briefly repeat the calculations of the paper [30]. The starting point is as follows. We do not know a true energy distribution D(E,m) but we do know the first moments of this distribution. In particular, we know the mean and the variance (3). Let us define the small parameter allowing us to expand the function D(E,m) in a perturbation theory series.

We present D(E,m) in the form

$$D(E,m)=\left(\begin{array}{l} N\\ n \end{array}\right)\exp\left[-N\varphi (m,E)\right],$$

where φ=φ(m,E) is an unknown function and use the Stirling formula to replace the summation in (2) by integration. Then, up to an insignificant constant, we obtain the partition function of the form

(5) $$Z\sim \int_{0}^{1}dm\int_{-\infty }^{\infty }dE\;e^{-NF(m,E)},$$

where

(6) $$\begin{array}{l} F(m,E)=S(m)+\beta (E-mH)+\varphi (m,E),\\ S(m)=-\ln2+\frac{1}{2}\left[\left(1+m\right)\ln\left(1+m\right)+\left(1-m\right)\ln\left(1-m\right)\right]. \end{array}$$

Let us estimate the integral (5) using the saddle point method. The equations for the saddle point are

(7) $$\frac{\partial F}{\partial m}=\frac{1}{2}\ln\left(\frac{1+m}{1-m}\right)+\frac{\partial \varphi }{\partial m}-\beta H=0,\;\frac{\partial F}{\partial E}=\frac{\partial \varphi }{\partial E}+\beta =0.$$

The solutions of these equations m=M and E=U are the spontaneous magnetization and the internal energy, respectively. Substituting these values in Equation (6), we obtain the free energy f(β)=F(M,U).

Now, we turn to defining the small parameter of the *m*-vicinity method. Since the magnetic field *H* does not influence the distribution D(E,m), we set here H=0. We write the function φ(m,E) as a perturbation theory series in the vicinity of point $E=E_{m}$:

(8) $$\varphi =\frac{1}{2}\varepsilon^{2}+\frac{1}{3!}\kappa_{3}\varepsilon^{3}+\frac{1}{4!}\kappa_{4}\varepsilon^{4}+\ldots ,\;\mathrm{where}\;\varepsilon =\frac{E-E_{m}}{\sigma_{m}}.$$

The quantities $\kappa_{k}$ up to a sign coincide with the semi-invariants of the distribution D(E,m) (see Appendix A).

The main idea of the *m*-vicinity method is the possibility to restrict ourselves by accounting for just a few first terms of the series (8). We can do this only if |ε|<<1 and in this case the *m*-vicinity method is sufficiently accurate. Let us clarify that we are not interested in the values of ε=ε(m,E) over the whole region of definition of the parameters m and E. We have to know the expansion (8) only in a small vicinity of the saddle point, which is close to the values m=M and E=U. Consequently, the small parameter we are looking for is

$$\varepsilon_{0}=\frac{U-E_{0}M^{2}}{\sigma_{0}(1-M^{2})}.$$

The smallness of the parameter $\varepsilon_{0}$ is the condition for applicability of the *m*-vicinity method.

In [30], there is a detailed analysis of the values of this parameter for different models. Here, we restrict ourselves with an estimate of $\varepsilon_{0}$ by means of the reverse-reasoning method. Suppose the parameter $\varepsilon_{0}$ is small and it is sufficient to use only the first term of the series (8) and set $\varphi =\varepsilon^{2}/2$. Then the second of the Equation (7) takes a simple form $\varepsilon =-\beta \sigma_{m}$ and we can rewrite the first of the Equation (7) as follows:

(9) $$\varepsilon \;\left(\varepsilon -\frac{E_{0}}{\sigma_{0}}\right)=-\frac{1-m^{2}}{4m}\ln\left(\frac{1+m}{1-m}\right).$$

We are interested in the behavior of the quantity $\varepsilon =\varepsilon_{0}$, where $\varepsilon_{0}$ is the root of Equation (9). The value of $\left|\varepsilon_{0}\right|$ reaches its maximum at the critical point $\beta =\beta_{c}$ (see [30]). In the limit m→0 ($\beta \to \beta_{c}$), Equation (9) takes the form

$$\varepsilon^{2}-\varepsilon \;\frac{E_{0}}{\sigma_{0}}+\frac{1}{2}=0.$$

The solution of this equation is

(10) $$\varepsilon_{\max}=-\frac{\left|E_{0}\right|}{2\sigma_{0}}+\frac{1}{2}\sqrt{\frac{E_{0}^{2}}{\sigma_{0}^{2}}-2},$$

and $\max\left|\varepsilon_{0}\right|=\left|\varepsilon_{\max}\right|$. Consequently, the *m*-vicinity method is applicable when the small parameter $\left|\varepsilon_{0}\right|\leq \left|\varepsilon_{\max}\right|$. According to Equation (10), it is necessary that $E_{0}^{2}/\sigma_{0}^{2}\geq 2$ or $\left|\varepsilon_{\max}\right|\leq 2^{-1/2}$.

A detailed analysis shows (see [27]) that a stricter inequality $E_{0}^{2}/\sigma_{0}^{2}\geq 8/3$ or $\left|\varepsilon_{\max}\right|\leq 6^{-1/2}$ defines the framework of the *m*-vicinity method. In another case, we obtain a jump of the spontaneous magnetization at the critical point, and this contradicts the known results.

We would like to mention here that when analyzing the dependence of our results on the lattice dimension d, we see that the ratio $E_{0}^{2}/\sigma_{0}^{2}$ depends on the effective number of the neighbors q introduced below in Equation (16). This parameter defines the number of interactions that we take into account. We can rewrite Equation (10) in terms of q; then $\varepsilon_{\max}=\sqrt{q}\left(-1+\sqrt{1-4/q}\right)/2\sqrt{2}$. From this equation, it follows that when q>>1 the value of $|\varepsilon_{\max}|∼1/\sqrt{2q}$. Consequently, when *q* increases, the value of the small parameter $\left|\varepsilon_{\max}\right|$ decreases quite rapidly and the accuracy of the *m*-vicinity method increases.

**Figure 1:**
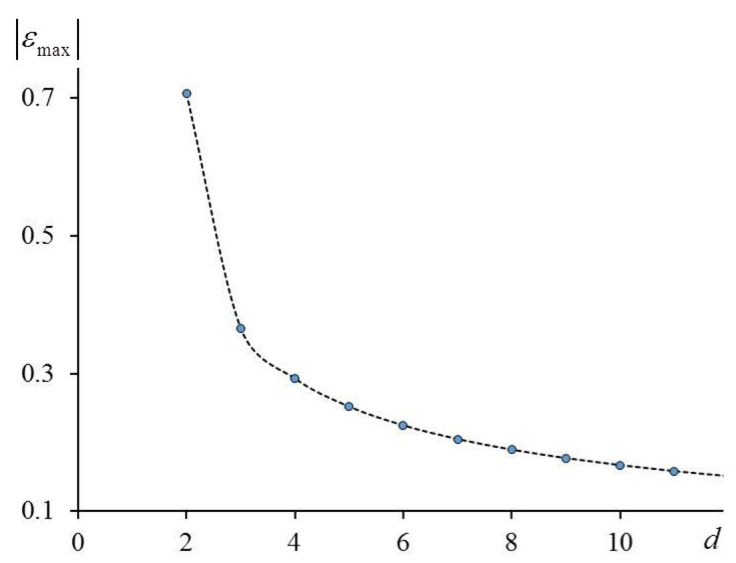
Small parameter $\left|\varepsilon_{\max}\right|$ as function of dimension of Ising model with interaction of nearest neighbors.

In Figure 1, we show the dependence of $\left|\varepsilon_{\max}\right|$ on d when we account for the nearest neighbors only (in this case $E_{0}^{2}/\sigma_{0}^{2}=d$). For a planar lattice, $\left|\varepsilon_{\max}\right|\approx 0.7$. Obviously, it is problematic to use it as a small parameter. Moreover, for a planar lattice, the inequality $E_{0}^{2}/\sigma_{0}^{2}\geq 8/3$ is not satisfied. Next, for a cubic lattice, the last inequality is satisfied and the value of $\left|\varepsilon_{\max}\right|\approx 0.36$ is more appropriate as a small parameter. Again, when d increases, the value of $\left|\varepsilon_{\max}\right|$ decreases, and the accuracy of the method increases accordingly.

Summing up, we can say that the Gaussian approximation is the first order of the perturbation theory in the small parameter $\left|\varepsilon_{\max}\right|$.

## 4. First Order of Perturbation Theory: Gaussian Approximation

In this section, we use the Gaussian approximation (4) for the distribution D(E,m). If in Equations (6)–(8) we set $\varphi =\varepsilon^{2}/2$, Equation (6) becomes

(11) $$F(m,E)=S(m)+\beta (E-mH)+\frac{1}{2}{\left(\frac{E-E_{m}}{\sigma_{m}}\right)}^{2};$$

the equations for the saddle point (7) are

(12) $$\frac{1}{2}\ln\left(\frac{1+m}{1-m}\right)=2m\beta \left(\left|E_{0}\right|+\varepsilon \sigma_{0}\right)+\beta H,\;\varepsilon =-\beta \sigma_{0}(1-m^{2}).$$

Eliminating the variable ε from these equations, we obtain an equation of state

(13) $$\frac{1}{2}\ln\left(\frac{1+m}{1-m}\right)=2m\beta \left[\left|E_{0}\right|-\beta \sigma_{0}^{2}(1-m^{2})\right]+\beta H.$$

This equation differs from the well-known equation of Bragg and Williams [29] by a term proportional to $\beta^{2}$. After a transformation of Equation (11), with the account for Equations (12) and (13), we obtain the expression for the free energy:

(14) $$f(\beta )=\frac{1}{2}\ln\left(\frac{1-m^{2}}{4}\right)+\beta \left|E_{0}\right|m^{2}-\frac{1}{2}\beta^{2}\sigma_{0}^{2}(1-m^{2})(1+3m^{2})$$

that defines f as a function of β and m. In Equation (14), the spontaneous magnetization m=m(β;H) is the solution of Equation (13). Again, by setting $\sigma_{0}^{2}=0$, we recover the known result from the mean field theory.

We would like to point out that the equality $E_{0}^{2}/\sigma_{0}^{2}=d$ is true only when we account for the interaction with the nearest neighbors. Then the inequality $E_{0}^{2}/\sigma_{0}^{2}\geq 8/3$ excludes the one- and two-dimensional Ising models from the consideration. Consequently, in this case, we can use the m-vicinity method only for the spatial dimensions d≥3.

### 4.1. Critical Point

In this subsection, we set H=0 and define the critical temperature under this assumption. For this purpose, we rewrite Equation (13) as

(15) $$\frac{1}{2m}\ln\left(\frac{1+m}{1-m}\right)=qb\left[1-b(1-m^{2})\right].$$

where we introduce dimensionless characteristics

(16) $$b=\beta \frac{\sigma_{0}^{2}}{\left|E_{0}\right|},\;q=\frac{2E_{0}^{2}}{\sigma_{0}^{2}}=\frac{{\left(\sum_{i,j=1}^{N}J_{ij}\right)}^{2}}{N\sum_{i,j=1}^{N}J_{ij}^{2}}.$$

The defined variables (16) are convenient since in Equation (15), the only parameter that depends on the type of the lattice is q.

When m→0 ($\beta \to \beta_{c}$), Equation (15) takes the form

(17) $$\frac{1}{q}=b(1-b).$$

Now, with the account of Equation (16), we obtain the expression for the critical temperature

(18) $$\beta_{c}=b_{c}\frac{\left|E_{0}\right|}{\sigma_{0}^{2}},\;\mathrm{where}\;b_{c}=\frac{1}{2}\left(1-\sqrt{1-\frac{4}{q}}\right).$$

**Figure 2:**
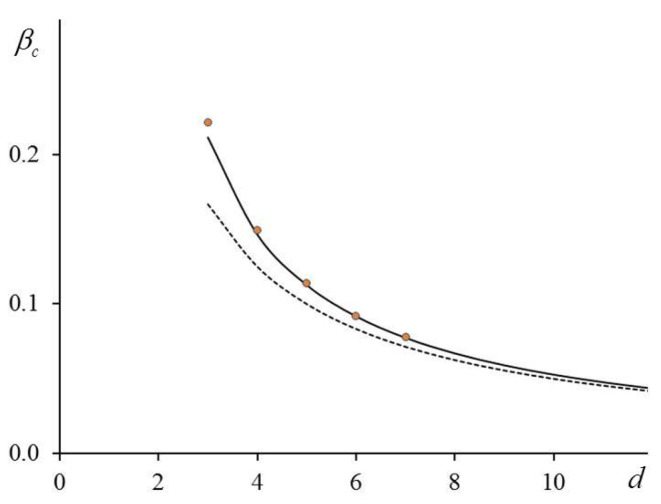
Critical temperature $\beta_{c}$ vs. d and account for interactions with nearest neighbors only: solid line corresponds to Equation (18); circles are simulations [9,21,23]; dashed line is result of mean field theory.

The critical value of $b_{c}$ depends only on the number q, and this parameter depends on the mean and the variance of the elements of the connection matrix. Since q is a characteristic of the interactions that we take into account, we regard it as an effective number of the neighbors. In particular, if we account for an isotropic interaction with the nearest neighbors only, $\beta_{c}=b_{c}$, q=2d, and d is the dimension of the lattice. Note that in this case, q is exactly equal to the number of spins with which the given spin interacts. Then Equation (18) describes pretty well the results of computer simulations for all the dimensions, which we examined (see Figure 2 and Table 1).

### 4.2. Analytical Expressions

Let us list the basic thermodynamic characteristics that we obtained from Equations (11)–(14).

(1) The interval $\beta <\beta_{c}$. When $\beta <\beta_{c}$ and m=0, from the above-mentioned equations, we obtain that the free energy, the internal energy, and the heat capacity are

(19) $$f=-\ln2-\frac{1}{2}\beta^{2}\sigma_{0}^{2},\;U=-\beta \sigma_{0}^{2},\;\mathrm{and}\;C=\beta^{2}\sigma_{0}^{2},$$

respectively.

(2) The interval $\beta \geq \beta_{c}$. To obtain the analytical expressions for the values f, U, and C, we solve Equation (15) for b:

(20) $$b=\frac{1}{2(1-m^{2})}\;\left[1-\sqrt{1-\frac{2(1-m^{2})}{qm}\;\ln\left(\frac{1+m}{1-m}\right)\;}\;\right]$$

and transform Expressions (12) and (14) to the forms

(21) $$f=S(m)-\frac{1}{2}qbm^{2}-\frac{1}{4}qb^{2}{\left(1-m^{2}\right)}^{2},$$

(22) $$U=E_{0}\left[m^{2}+b{\left(1-m^{2}\right)}^{2}\right],$$

(23) $$\sigma_{E}^{2}=\sigma_{0}^{2}{\left(1-m^{2}\right)}^{2}+4E_{0}^{2}\frac{m^{2}\left(1-m^{2}\right){\left[1-2b\left(1-m^{2}\right)\right]}^{2}}{1-qb\left(1-m^{2}\right)\left[1-b\left(1-3m^{2}\right)\right]}.$$

Here, $\sigma_{E}^{2}=-d^{2}f/d\beta^{2}$ is the energy variance that is related to the heat capacity via $C=\beta^{2}\sigma_{E}^{2}$. The expression (23) is a result of differentiating the second of Equation (12) with respect to variable β.

If we regard the magnetization as an independent variable m∈[0,1] and consider the values of f, U, and $\sigma_{E}^{2}$ as functions of *m*, then Equations (20)–(23) define implicitly the dependence of f, U, and $\sigma_{E}^{2}$ on the inverse temperature $\beta =b\left|E_{0}\right|/\sigma_{0}^{2}$ at the interval $\beta \geq \beta_{c}$.

### 4.3. Critical Parameters

To examine the behavior of the thermodynamic characteristics near the critical point, we introduce a relative inverse temperature

$$t=\frac{\beta -\beta_{c}}{\beta_{c}}.$$

Omitting intermediate calculations, we only present the most important critical dependences.

(1) From Equations (19)–(23), it follows that at $\beta =\beta_{c}$ the free energy and the internal energy are continuous functions, and the heat capacity has a jump. Indeed, when $\beta >\beta_{c}$ Equation (23) holds, and in the limit m→0 ($\beta \to \beta_{c}$) we obtain

(24) $$\sigma_{E}^{2}=\sigma_{0}^{2}+6E_{0}^{2}\frac{{\left(1-2b_{c}\right)}^{2}}{1-3qb_{c}^{2}},$$

where $b_{c}$ is defined by Equation (18). Comparing this expression with Equation (19), we see that at $\beta =\beta_{c}$ the energy variance has a jump and consequently the heat capacity also has a jump:

(25) $$\Delta C=\frac{3}{2}q^{2}b_{c}^{2}\frac{{\left(1-2b_{c}\right)}^{2}}{1-3qb_{c}^{2}}.$$

For the lattice dimensions *d* = 5, 6, and 7, the authors of [23] used computer simulations to estimate the jumps of the heat capacity. The comparison of their results with the values following from Equation (25) shows:

$$\begin{array}{l} d=5,\;\;\Delta C^{\left(\exp\right)}=1.8703,\;\;\Delta C^{\left(theor\right)}=1.8469\\ d=6,\;\;\Delta C^{\left(\exp\right)}=1.7403,\;\;\Delta C^{\left(theor\right)}=1.7394\\ d=7,\;\;\Delta C^{\left(\exp\right)}=1.6860,\;\;\Delta C^{\left(theor\right)}=1.6824. \end{array}$$.

We see that Equation (25) provides very good agreement with the computer simulations; the larger d is, the better this agreement.

(2) When $\beta >\beta_{c}$, the value of the spontaneous magnetization near the critical point (t→0) obtained from Equation (15) is

(26) $$M=A\sqrt{t},\;A={\left(\frac{1-qb_{c}^{2}}{\frac{1}{3}-qb_{c}^{2}}\right)}^{\frac{1}{2}}.$$

This expression differs from the dependence $M∼t^{\frac{1}{8}}$ that is valid for the two-dimensional Ising model (*q* = 4); however, it qualitatively coincides with the expressions $M=2\sqrt{t}$ and $M=\sqrt{3\;t}$ obtained in the framework of the van der Waals theory [13] and the mean field theory, respectively. Note that our result tends to the result of the mean field theory in the limit q>>1. It is also worthwhile to note that Equation (26) predicts a larger magnetization than the mean field theory, $A>\sqrt{3}$ for any q≥4. If q≤11 (A>2), the van der Waals magnetization is less than the value (26) and when q>11 (A<2), it is larger than the value (26).

(3) From Equation (13), it follows that at the critical point, the susceptibility has a jump

(27) $$\chi^{-1}=\{\begin{array}{c} \begin{array}{l} -q\;t\;\sqrt{1-4/q}\;,\;t<0 \end{array}\\ \;2q\;t\;\sqrt{1-4/q}\;,\;t>0. \end{array}$$

Comparing with the analogous expression of the mean field theory, we see that an extra factor $\sqrt{1-4/q}$ appears in Equation (27), and it tends to 1 when q>>1. As opposed to the mean field theory [13], in Equation (27), q is the effective number of the neighbors (16).

(4) It is easy to see that our model satisfies the similarity hypothesis. Indeed, when we expand expression (13) in small parameters, m and t, we obtain the dependence H=H(m, t) that can be rewritten in the classical form $\beta_{c}H=m{\left|m\right|}^{\delta -1}h_{s}(tm^{-2})$ with the critical exponent δ=3 and the scaling function

(28) $$h_{s}(x)=\left(\frac{1}{3}-qb_{c}^{2}\right)-x\left(1-qb_{c}^{2}\right).$$

### 4.4. Magnetization Distribution

The integral

(29) $$P(m)=Z^{-1}\int_{E_{0}}^{\left|E_{0}\right|}D(E,m)dE$$

defines the probability of finding the system in a state with the magnetization *m*. In the Gaussian approximation we use here, D(E,m) is defined by Equation (4). We estimate this integral with the aid of the saddle point method. The value of P(m) is accurate to a normalization constant $P_{0}$

(30) $$P(m)=P_{0}\;e^{-N\Phi (m)},\;\mathrm{where}\;\Phi (m)=S(m)+\beta E_{m}-\frac{1}{2}\beta^{2}\sigma_{m}^{2}.$$

**Figure 3:**
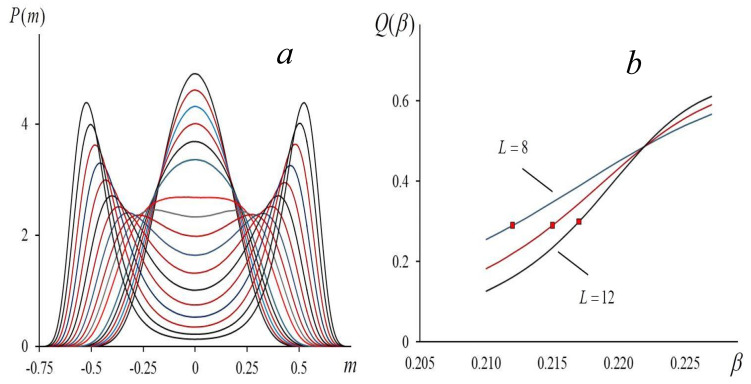
Magnetization: (**a**) distribution of magnetization P(m) for three-dimensional Ising model (q=6, L=12) for different inverse temperatures β=0.210, 0.211, …, 0.227; (**b**) dependence of Binder cumulant on β for three-dimensional lattices with *L* = 8, 10, and 12. Markers show values of β where bimodal distribution replaces unimodal.

In Figure 3a, we show the typical behavior of the curves (30). As we might expect, after crossing the critical point, the bimodal distribution replaces the unimodal distribution. We can use Equation (30) when analyzing the Binder cumulant $Q=1-\langle m^{4}\rangle /3{\langle m^{2}\rangle }^{2}$ (see [31]). In Figure 3b, we show the curves Q=Q(B) for cubic lattices whose linear sizes are L= 8, 10, and 12. We are interested in the value of the cumulant $Q_{c}=Q(\beta_{c})$ at the critical point. To calculate $Q_{c}$ we use the Taylor expansion of the function Φ(m) in Equation (30):

(31) $$\Phi (m)\approx -\frac{1}{2}\beta^{2}\sigma_{0}^{2}+\frac{1}{2!}a_{2}m^{2}+\frac{1}{2!}a_{4}m^{4},$$

where

$$a_{2}={\frac{d^{2}\Phi }{dm^{2}}|}_{m=0}=1+2\beta E_{0}+2\beta^{2}\sigma_{0}^{2},\;a_{4}={\frac{d^{4}\Phi }{dm^{4}}|}_{m=0}=1-12\beta^{2}\sigma_{0}^{2}.$$

This expansion is useful when $\beta \leq \beta_{c}+O\left(N^{-1/2}\right)$ and the value of P(m) is noticeably nonzero only when m<<1. In this case, the distribution (30) takes the form

$$P(m)=P_{0}\exp\left[-\frac{1}{2!}Na_{2}m^{2}-\frac{1}{4!}Na_{4}m^{4}\right]$$

which allows us to calculate the normalization constant $P_{0}$ easily as well as the mean values $\langle m^{2}\rangle$ and $\langle m^{4}\rangle$. It is not difficult to see that when $\left|\beta -\beta_{c}\right|>O\left(N^{-1/2}\right)$, we can consider the distribution P(m) as purely Gaussian in full agreement with the results of [31]. However, when $\beta \to \beta_{c}$ we have $a_{2}\to 0$ and at the critical point, the distribution takes the form $P(m)=P_{0}\exp\left[-Na_{4}m^{4}/4!\right]$. Using this expression to calculate the critical value of the Binder cumulant [31] in the limit N→∞ we obtain

(32) $$Q_{c}=1-\frac{\Gamma (5/4)\cdot \Gamma (1/4)}{3\Gamma {\left(3/4\right)}^{2}}\approx 0.2705.$$

It is interesting that in the limit N→∞ the value $Q_{c}$ does not depend on any parameters of the model (the lattice type, the character of the long-range interaction, and so on). Although we obtained this result in the framework of the Gaussian approximation (4), it has a general character. Indeed, let D(E,m) be an unknown function, and integration (29) results in expression (30) where Φ(m) is also an unknown function. By a symmetry argument, it follows that only even powers of *m* are present in the Taylor expansion of this function. At the critical point, the second derivative of function Φ(m) equals to zero (the bimodal distribution replaces the unimodal) and the Taylor series starts from the term $\sim m^{4}$. We assume that the function Φ(m) has no singularities and its derivatives are finite. Then, we can leave only the term $\sim m^{4}$ in the exponent of the distribution P(m). The reason is that when integrating over m the account for the terms of the higher orders leads to corrections of the order $N^{-1/2}$. In other words, we can present the magnetization distribution as $P(m)=P_{0}\exp\left[-Na_{4}m^{4}/4!\right]$ where $a_{4}$ is an unknown, which is canceled out in the calculation of $Q_{c}$ and does not influence the final form of expression (32).

### 4.5. Density of States

In Section 4.2, we derived Equations (19)–(23), which allowed us to obtain implicitly the logarithmic density of states

**Figure 4:**
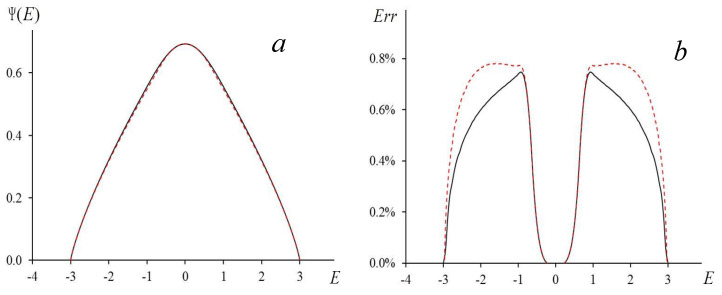
(**a**) Logarithmic density of states: solid line—Wang-Landau algorithm for cubic lattice of size *L* = 40; dashed line—calculation by Formulas (19)–(23) and Legendre relations. (**b**) Solid line is modulus of relative error for calculations when using Legendre relations; dashed line is relative error of Equation (34).

Ψ(E)=βE−f(β); E=df/dβ

using the Legendre relations. When β changes from 0 to ∞, the value of E changes from 0 to $E_{0}$ and for each β we obtain a pair of values E and Ψ(E). In such a way, we generate the function Ψ(E), which we suppose to be symmetric: Ψ(−E)=Ψ(E). In Figure 4a, we present the comparison of our results with computer simulations.

Let us determine an explicit form of the dependence Ψ=Ψ(E). The integral

$$D(E)=\int_{-1}^{1}D(E,m)dm\approx c_{1}\cdot \int_{-1}^{1}e^{-N\Lambda (m,E)}dm=c_{2}e^{N\Psi \left(E\right)}$$

defines the density of states or, in other words, the number of states with the energy E. Here $\Lambda (m,E)=S(m)+{\left(E-E_{m}\right)}^{2}/2\sigma_{m}^{2}$; $c_{1}$ and $c_{2}$ are non-essential constants. Therefore, the logarithmic density of states is $\Psi (E)=\Lambda (m_{E},E)$, where $m_{E}=m_{E}(E)$ is the saddle point, which is a solution of the equation ∂Λ(m,E)/∂m=0. After some transformations, we can write this equation as

(33) $$\frac{{\left(1-m_{E}^{2}\right)}^{3}}{2m_{E}}\ln\left(\frac{1+m_{E}}{1-m_{E}}\right)=q\left(1-\frac{E}{E_{0}}\right)\left(\frac{E}{E_{0}}-m_{E}^{2}\right).$$

From this equation, it follows that

(34) $$\Psi (E)=\{\begin{array}{ccc} \ln2-\frac{E^{2}}{2\sigma_{0}^{2}}, & \mathrm{when} & E\geq E_{0}b_{c}\\ -S(m_{E})-\frac{1}{2\sigma_{0}^{2}}{\left(\frac{E-E_{0}m_{E}^{2}}{1-m_{E}^{2}}\right)}^{2}, & \mathrm{when} & E<E_{0}b_{c}, \end{array}.$$

where $b_{c}$ is defined by Equation (18) and

(35) $$m_{E}^{2}\approx 1-2r\cdot \cos\varphi_{E},\;r=\sqrt{\frac{q}{3}\left(1-\frac{E}{E_{0}}\right)},\;\mathrm{and}\;\varphi_{E}=\frac{1}{3}\left[\pi +\arccos\left(\frac{9r}{2q}\right)\right].$$

Equation (35) is an approximate result obtained taking into account that the left-hand side of Equation (33) contributes significantly to the solution of this equation only when $m_{E}<<1$. Under this condition $\ln\left[\left(1+m_{E})/(1-m_{E}\right)\right]/2m_{E}\approx 1$ Equation (33) reduces to a cubic equation for the quantity $\left(1-m_{E}^{2}\right)$. The solution of this equation has the form (35). The approximate solution (34) differs by fractions of a percent from the exact solution obtained by means of the Legendre relations (see Figure 4b).

## 5. Second Order of Perturbation Theory: Account for Third Moment

In this section, we analyze what happens if, when approximating the distribution D(m,E), we account for the third moment. We examine only the simplest case, supposing that all the nonzero elements of the connection matrix are equal ($J_{ij}=J$). Otherwise, the obtained expressions are too cumbersome, and it is very difficult to analyze them. We restrict ourselves only by first two terms of the series expansion (8) and set

(36) $$\varphi =\frac{1}{2}\varepsilon^{2}+\frac{1}{3!}\kappa_{3}\varepsilon^{3},\;\mathrm{where}\;\varepsilon =\frac{E-E_{m}}{\sigma_{m}}.$$

We define the coefficient $\kappa_{3}$ from the following considerations. It is necessary that the third moment of our approximation exp[−N⋅φ(m,E)] coincides with the third moment of the true distribution D(m,E): $\int {\left(E-E_{m}\right)}^{3}\exp\left[-N\cdot \varphi (m,E)\right]dE=\mu_{3}(m)$. In the Appendix A, we show that for this, it is necessary that the coefficient $\kappa_{3}$ is equal to the third semi-invariant of the distribution D(m,E): $\kappa_{3}=-\mu_{3}(m)/\sigma_{m}^{3}$. In the Appendix A, we also obtain the expression for the third moment of the distribution D(m,E), which is equal to $\mu_{3}(m)=-2qm^{2}{\left(1-m^{2}\right)}^{2}$. Here q is the effective number of the neighbors (see Equation (16)).

Then

$$\kappa_{3}=\frac{2qm^{2}}{\sigma_{0}^{3}(1-m^{2})},$$

and expression (6) for the function F(m,E) takes the form (H=0):

$$F(m,E)=S(m)+\beta E+\frac{1}{2}\varepsilon^{2}+\frac{1}{3!}\kappa_{3}\varepsilon^{3}.$$

We recall that $E_{m}=E_{0}m^{2}$, $\sigma_{m}=\sigma_{0}(1-m^{2})$, $E_{0}=-q/2$, and $\sigma_{0}=\sqrt{q/2}$.

The system of equations that define the saddle point has the form

(37) $$\begin{array}{l} \frac{\partial F}{\partial E}=\beta +\frac{\varepsilon +\kappa_{3}\varepsilon^{2}/2}{\sigma_{m}}=0,\\ \frac{\partial F}{\partial m}=\frac{1}{2}\ln\frac{1+m}{1-m}+\left(\varepsilon +\frac{1}{2}\kappa_{3}\varepsilon^{2}\right)\dot{\varepsilon }+\frac{{\dot{\kappa }}_{3}}{3!}\varepsilon^{3}=0, \end{array}$$

where $\dot{\varepsilon }=\partial \varepsilon /\partial m$. The first of the equations of (37) provides the relation

(38) $$\varepsilon +\kappa_{3}\frac{\varepsilon^{2}}{2}=-\beta \sigma_{m}.$$

Moreover, by direct calculations, we obtain

$$\dot{\varepsilon }=\frac{2m}{1-m^{2}}\left(\varepsilon +\frac{\left|E_{0}\right|}{\sigma_{0}}\right),\;{\dot{\kappa }}_{3}=\frac{4qm}{\sigma_{0}\sigma_{m}^{2}},$$

and substituting these equalities in the second Equation (37), we finally have

(39) $$\frac{1}{2m}\ln\frac{1+m}{1-m}=2\beta \left(\varepsilon \sigma_{0}+\left|E_{0}\right|\right)-\frac{2q}{3\sigma_{0}\sigma_{m}^{2}}\varepsilon^{3}.$$

Generally speaking, for deriving an equation relating m and β it is necessary to solve Equation (38) and to determine ε=ε(m) and substitute it into Equation (39). Analyzing this equation, it would be possible to define the region of applicability of the *m*-vicinity approximation with account for the third moment and to obtain an expression for the critical temperature. It is rather difficult to solve this problem analytically. This is the reason why we restrict ourselves by analyzing the influence of the third moment $\mu_{3}(m)$ on the value of the critical temperature defined by Equation (39) when m=0.

When m→0, from Equation (38) it follows that $\varepsilon \to -\beta \sigma_{0}$. Then the second term in the right-hand side of Equation (39) tends to $-2q\beta^{3}/3$ and this equation itself takes the form $1=2\beta \left(-\beta \sigma_{0}^{2}+\left|E_{0}\right|\right)+2q\beta^{3}/3$. By virtue of the expressions for $E_{0}$ and $\sigma_{0}$ we obtain the equation for the critical value of the inverse temperature $\beta_{c}$:

(40) $$\frac{1}{q}=\beta -\beta^{2}+\frac{2k}{3}\beta^{3},\;\mathrm{where}\;k=1.$$

If in this equation we set k=0, we obtain Equation (17) that corresponds to the above-discussed case of the Gaussian approximation. We introduce the parameter k since in what follows, we will use it as a fitting parameter.

Solving Equation (40) for d∈[3,7] numerically, we see the worse agreement of the obtained results with the computer simulations (see Table 1). Previously (see [24], Section 17.6) it was mentioned that an account for higher moments does not always lead to an increase in the quality of an approximation. You can expect such a result only for a narrow class of distributions. This question was under analysis when studying the Gram–Charlier and the Edgeworth series expansions [24,25]. In particular, it turned out that when we omit the higher order terms of the infinite series (8), the error is of the order of the first omitted term. To compensate this error, we use the fitting parameter k. The author of [24] (s.17.6) suggests using this receipt if the optimal value of *k* gives a better agreement with all the numerical values. The questions of convergence of an infinite series are of secondary importance when solving a specific problem.

With those arguments in mind, we found that an agreement with the best numerical results is better when k<0. In this case, the solution of Equation (40) takes the form

(41) $$\beta_{c}=B\cos\phi -\frac{1}{2\left|k\right|},$$

where

$$B=\frac{\sqrt{1+2|k|}}{\left|k\right|},\;\phi =\frac{1}{3}\left(2\pi -\arccos R\right),R=-\frac{1}{(1+2|k|{)}^{3/2}}\left(1+3|k|+\frac{6k^{2}}{q}\right).$$

For *d* = 3, the best agreement is reached when k=−0.8. It turned out that this value of *k* is optimal for all examined dimensions: the solutions of Equation (41) with the same fitting parameter provide a very good agreement with the computational data for the lattices of all the dimensions 3≤d≤7 (q=2d). As we see from Table 1, accounting for the third moment and introduction of the fitting parameter k=−0.8 reduce the relative error

$$Err=100\%\cdot \left(\frac{\beta_{c}^{\left(\exp\right)}-\beta_{c}^{\left(theor\right)}}{\beta_{c}^{\left(\exp\right)}+\beta_{c}^{\left(theor\right)}}\right)$$

by 1 to 2 orders of magnitude, and it becomes comparable with the computational error. When we account for the interactions with the second and third neighbors, the introduction of the third moment also improves the agreement with the computer simulations significantly (see the next section).

## 6. Comparison with Computer Simulations: Three-Dimensional Ising Model

To estimate the accuracy and the correctness of the obtained equations, we performed computer simulations using the Metropolis algorithm and the algorithm of Wang and Landau [11]. We restricted ourselves to the examination of the three-dimensional Ising model supposing that for lattices of higher dimensions (d≥4), the agreement with the numerical results would be only better. In the course of our simulations, we explored the functions f=f(β), U=U(β), and C=C(β). This allowed us to define the dependence of the critical temperature $\beta_{c}=\beta_{c}(q)$ on q, to calculate the logarithmic density of states Ψ(E), and to analyze how the magnetization distribution changed when the inverse temperature increased.

Recently, there is a strong interest in an account for interactions with the second and the third neighbors (see, for example, [32,33]). However, we do not know any analytical estimates for the critical temperatures. Our Equation (18) is quite accurate for such problems too.

We use the Metropolis algorithm to examine the three-dimensional lattices of the linear sizes L = 20, 32, and 64 with periodic boundary conditions. We look for the dependences of the critical parameters on the effective number of neighbors q. We suppose that all the interaction constants with the 6 nearest neighbors are equal to one ($J_{NN}=1$). We start with varying the value of the constant of interaction with the 12 next-nearest neighbors $J_{NN}$ from 0 to 1, and this means that q changes from 6 to 18. Then we fix the value $J_{NNN}=1$ and vary the interaction constant with the 8 next-next-nearest neighbors $J_{NNNN}$ from 0 to 1 so that q changes from 18 to 26. The initial state of the system is random. To bring the system to a state close to equilibrium for a given temperature, at the first $10^{4}\cdot L^{3}$ Monte Carlo steps, we do not accumulate the statistical data. We flip spins according to the Metropolis algorithm in the order of their sequence in the lattice. After each flip, we measure the values of the energy and the magnetization. The total number of the Monte Carlo steps is $4\cdot 10^{5}\cdot L^{3}$. We vary the inverse temperature with the step size $\Delta \beta \approx 1.8\cdot 10^{-4}$ for L=20, with the step size $\Delta \beta \approx 7.5\cdot 10^{-6}$ for L=32, and with the step size $\Delta \beta \approx 10^{-4}$ for L=64. We define the critical point as the point of maximum of the energy variance $\sigma_{E}^{2}=\sigma_{E}^{2}(\beta )$. At this point we fix the values of $U_{\max}=U(\beta_{\max})$, $\sigma_{\max}^{2}=\sigma_{E}^{2}(\beta_{\max})$ as well as other values. We also use the Metropolis algorithm to calculate the energy and magnetization moments near the critical temperatures. In Table 2, we present the numerical data for L=64.

This calculation had two goals. The first was to analyze the role of the next terms of the expansion series (8). The second goal was to examine the influence of the account for the next-nearest and the next-next-nearest neighbors on the character of the dependence C=C(β) and find if the type of the singularity at the critical point changed to a finite jump.

### 6.1. Dependence $\beta_{c}=\beta_{c}(q)$

**Figure 5:**
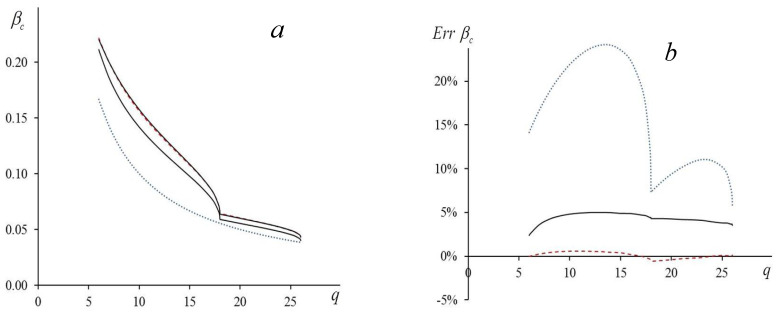
(**a**) Dependence of critical temperature $\beta_{c}=\beta_{c}(q)$ on effective number of neighbors q for three-dimensional Ising model: upper solid line presents computer simulation data for L=64; dashed line that merges with upper solid line is calculation with account for third moment (see Equation (42)); lower solid curve corresponds to Gaussian approximation (see Equation (18)); dotted line is $\beta_{c}$ that follows from mean field theory. (**b**) Relative error: lower dashed line corresponds to account for third moment (42); solid line corresponds to Gaussian approximation (18); upper dotted line corresponds to mean field theory.

In Figure 5a, we show the dependence of $\beta_{c}$ on q. We compare the numerical values (the upper solid line) with those that follow from Equation (18) for the Gaussian approximation (the lower solid line). As we see, the solid curves have similar shapes, and when we scale the theoretical curve by a factor of ~1.06, they practically merge. Such a coincidence cannot be accidental. From Equation (18) it follows that in the Gaussian approximation, the curve $\beta_{c}(q)$ has singularities at q=18 and q=26:

$${\frac{d\beta_{c}}{dq}|}_{\;q\to 18-0}=-\frac{\beta_{c}}{6\sqrt{\;18-q}}\;\mathrm{and}\;{\frac{d\beta_{c}}{dq}|}_{\;q\to 26-0}=-\frac{\beta_{c}}{3\sqrt{\;26\;(26-q)}}.$$

The curve obtained by means of computer simulations shows the same singularities. In our simulations, we check this statement very accurately, changing q in the vicinities of these points with a very small step size ($\Delta q\sim 10^{-3}$).

Agreement of the numerical results with our theory becomes much better when we account for the third moment in Equation (36) and solve numerically the equation for the saddle point (37) (accounting for Equations (A4) and (A11) of the Appendix A). In this case, the relative error does not exceed a fraction of a percent. When we account for interactions beyond the nearest neighbors, the expression for the third moment $\mu_{3}(m)$ becomes so cumbersome that it does not allow us to obtain analytical expressions for the critical temperature analogous to Equations (40) and (41). On the other hand, we can use a simple empirical formula

(42) $$\beta_{c}=\frac{\beta_{c}^{\left(0\right)}}{1+0.207\cdot (12J_{NNN}+8J_{NNNN})},$$

where $\beta_{c}^{\left(0\right)}\approx 0.22165$ is the critical value given by formula (41), accounting for the nearest neighbors only ($J_{NNN}=J_{NNNN}=0$). Comparing the solid and dashed lines in Figure 5b, we see that expression (42) describes the computer simulations much better than formula (18)—in comparison with the Gaussian approximation, the relative error decreases by an order of magnitude; it decreases nearly by two orders of magnitude comparing with the mean field theory.

### 6.2. Dependence C=C(β,q)

Our analysis shows that in the case of a three-dimensional Ising model, our account for the next-nearest and the next-next-nearest neighbors does not qualitatively change the behavior of the heat capacity at the critical point. Next, when the number of spins *N* increases, the peak of the curve C=C(β,q) only increases and the character of the singularity remains the same for all 6≤q≤26.

The dependences of the heat capacity on the critical temperature calculated numerically differ significantly from the ones defined by Equations (19) and (23). This means that we cannot use Equations (21)–(23) to describe the dependences C=C(β,q). Equation (25) that defines the finite jump of the heat capacity and works well when d>4 is not applicable in the three-dimensional case.

**Figure 6:**
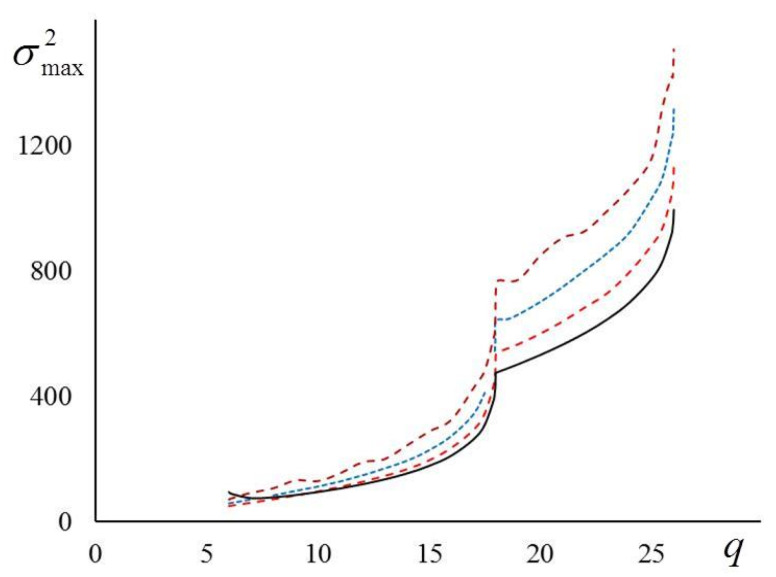
Dependence $\sigma_{\max}^{2}$ vs. q: solid line—Equation (24); dashed lines—computer simulations for L=20, 32, 64 (bottom-to-top).

In Figure 6, we show the dependences of the maximal energy variance $\sigma_{\max}^{2}=\sigma_{\max}^{2}(q)$ on the effective number of neighbors. From the figure it is obvious that, first, the energy variance at the critical point increases when q increases. Second, we see that with an increase in the lattice size L the differences between the computer simulations and the theoretical curve grow quickly. Nevertheless, the dependences of $\sigma_{\max}^{2}=\sigma_{\max}^{2}(q)$ on q repeat qualitatively the theoretical curve (compare solid and dashed lines in Figure 6).

The situation with the lattices of larger dimensions d is quite the opposite. Let us discuss it here shortly. The larger the dimension of the lattice, the better the theoretical expressions (21)–(23) describe the numerical results for the heat capacity in the vicinity of the critical point. When d≥5, the agreement is good, both qualitatively and quantitatively.

**Figure 7:**
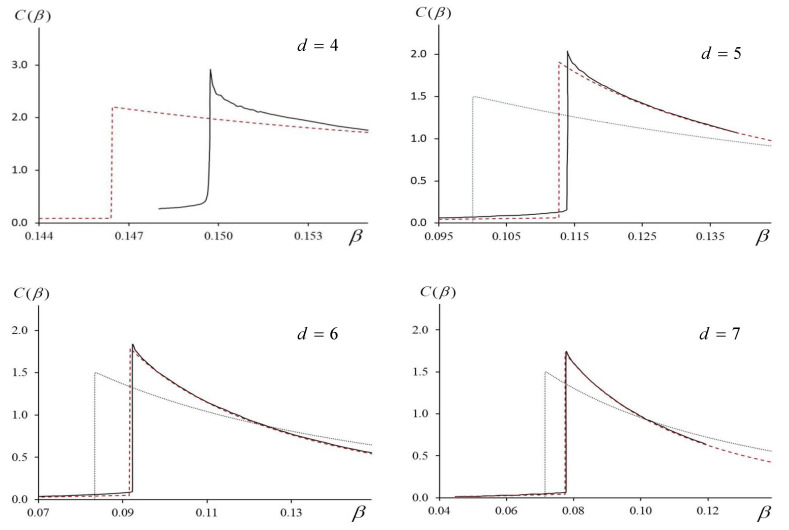
Heat capacity C vs. β for d = 4, 5, 6, 7. Computer simulations—solid lines; theory (Equations (19) and (23))—dashed lines. Data for d=4 and L=80 are from [21]; curves for d = 5, 6, and 7 are numerical results [23]. Dotted line in figures for d = 5, 6, 7 represents mean field theory.

In Figure 7, the dashed lines are the theoretical curves C=C(β) (Equations (21)–(23)); the solid lines are the results of computer simulations. For d=4, the value of L=80 (see [21]), and for the dimensions d=5,6 and 7, we use the data from [23]. We see that our formulas describe the numerical results pretty well. When d increases, the agreement of the theory and the simulations improves notably; when d=7 the theoretical and the numerical curves almost merge. In the same figure, we also present the graph for d=4; however, to date, there is no real understanding of what happens in the four-dimensional case. We do not know for sure if there is a jump of the heat capacity or an infinite singularity. In the same figures, we show the curves obtained in the framework of the mean field theory. We see that our approximation describes the results of the numerical experiments much better.

### 6.3. Magnetization Distribution P(m) and Logarithmic Density of States

With the aid of the Metropolis algorithm, we calculated the magnetization distribution near the critical temperature for the three-dimensional Ising model, taking into account the nearest neighbors only (L = 8, 10, and 12). To increase the accuracy of the Monte Carlo method, we performed $3\cdot 10^{7}\cdot L^{3}$ steps. To fix the inverse temperature where the bimodal distribution replaces the unimodal, we used the step size $\Delta \beta =10^{-3}$. For L=12, we present the curves of the magnetization distribution in Figure 3a. The obtained distributions allow us to calculate the Binder cumulants Q(β) (see [10,31]). In Figure 3b, the curves Q=Q(β) intersect at a point β=0.222, Q=0.488. This value of β defines the critical temperature for the infinite lattice. At the curves in Figure 3b, the square markers are the points where the bimodal distributions replace the unimodal. At these points, the cumulants are approximately equal to 0.29, and this value is sufficiently close to the value predicted by Equation (32). When L increases, these points shift to the point of the curves intersection that corresponds to the critical value $\beta_{c}$ in the asymptotic limit L→∞.

To obtain the density of states, we used the algorithm of Wang and Landau [11]. We performed the calculations for the cubic lattice of the size L=40 without parallel computing and only accounting for the interaction with the nearest neighbors. As a criterion of the flatness of the histogram of visited states, we adopted the condition that all the values of the histogram had to be larger than 80% of its mean value. When this condition was satisfied, the algorithm reduced the modification factor according to the formula $f_{i+1}^{\left(\mathrm{mod}\right)}=\sqrt{f_{i}^{\left(\mathrm{mod}\right)}}$. The simulations stopped when the modification factor became less than $f_{final}^{\left(\mathrm{mod}\right)}=\exp(10^{-10})$.

In Figure 4a, we present the graphs of the logarithmic density of states calculated numerically as well as the ones based on the equations of Section 2. The density of states calculated using Equations (19)–(23) and the Legendre relations was in a good agreement with the numerical data. We see that the maximal obtained error, which is about 0.7%, corresponds to the critical energy $E=U_{c}$. The results of the approximate formula (34) are somewhat less accurate; however, in these calculations, the deviation from the numerical results is also less than 0.8% (see Figure 4b).

## 7. Discussion

We used the *m*-vicinity method to investigate the Ising model on d-dimensional hypercube lattices for 3≤d≤7. (We recall that this method is not applicable for the lattices of lower dimensions.) The *m*-vicinity $\Omega_{m}$ consists of all the configurations of the same magnetization *m*. We based our method on the series expansion of the logarithm density of states in the *m*-vicinities (Section 2). In the main part of the paper, we discuss the interaction with the nearest neighbors. Only in Section 6, for a cubic lattice, we analyzed both the short-range and long-range interactions.

When we account only for an isotropic interaction of the nearest neighbors, the small parameter is $\left|\varepsilon_{\max}\right|=\sqrt{d}\left(1-\sqrt{1-2/d}\right)/2$ (see Equation (10)). The value of this parameter decreases from 0.366 when d=3 to 0.159 when d=7 (see Figure 1). Not surprisingly, the agreement between the theoretical results and computer simulations becomes better when the dimension of the lattice increases.

Summing up, we would like to list the main features of the *m*-vicinity method.

(1) The first order of the perturbation theory is equivalent to the Gaussian approximation of the true density of states in the *m*-vicinities supposing that the first two moments of the density of states and its Gaussian approximation coincide.

In this case, we obtained the simple analytical expression (18) for the critical value of the inverse temperature, which described quite accurately the results of computer simulations for different lattices (see Figure 2). When d=3, the relative error between the theoretical estimate $\beta_{c}$ and the computer simulations is 2.39%. When d increases, the relative error decreases to 0.18% for d=7 (see Table 1).

In the framework of the same approximation, we obtained analytical expressions (19)–(23) that define implicitly the dependence of the free energy and its derivatives on the temperature. Based on these results, we calculated the dependence of the heat capacity C(β) on the inverse temperature β. As it follows from Figure 7, for d≥5, the obtained graphs match the curves obtained numerically [23]. The values of the critical exponents that follow from Equations (25)–(28) coincide with the known mean field results. For d≥5, Equation (25) predicts a finite jump of the heat capacity whose value is only 0.6% less than the value obtained by computer simulations [23].

(2) The second order of the perturbation theory requires going beyond the Gaussian approximation and accounting for the third moment in the expansion (36). We suppose that the first three moments of the approximate and the true distributions coincide. However, this does not automatically improve the agreement of the theoretical estimates of the critical temperature and its numerical values. The reason is an irregular convergence of the Gram–Charlier and the Edgeworth series expansions [24]. The agreement improves significantly when we introduce the fitting parameter that is the same for all the dimensions d (see Section 5). After that, the second order perturbation Formula (41) describes the computer simulations within a fraction of the percent (see Table 1). From Figure 5, it follows that the same is true when we also account for the interactions with the next-nearest and the next-next-nearest neighbors.

(3) The results of our analysis show that when the dimension of the Ising model d≥5, the *m*-vicinity method describes the properties of the system pretty well, both qualitatively and quantitatively. When d=4, the type of the singularity of heat capacity still remains unclear [20,22]. Consequently, the question about the applicability of the *m*-vicinity method in this case remains open.

In the case d=3, the approach discussed here is incorrect because it predicts a finite jump of the heat capacity at the critical point and the classical values of critical exponents. This result contradicts the widely accepted concept of the singularity at the critical point in the modern phase-transitions theory. Nevertheless, this method allows one to calculate the critical temperature quite accurately (see Table 1) as well as to describe its dependence on the number of the neighbors (see Figure 5 and Equations (41) and (42)). We conclude that when d=3 our theory provides good results for the dependences of the free energy and the logarithmic density of states but not for their derivatives. Indeed, when for d=3 we use Equations (34) and (35) to calculate the logarithmic density of states, the result is in a good agreement with the computer simulations data. From Figure 4 we see a notable deviation from the numerical curve (~0.7%) only in a narrow vicinity of the point $E=U_{c}$.

(4) Finally, we would like to note that a good agreement of the theoretical results for the density of states Ψ=Ψ(E) with the data of computer simulations allows us to use the obtained expressions as an initial approximation for the Wang-Landau algorithm. We hope that our results will allow one to speed the algorithm up and to increase its accuracy.

## Figures and Tables

**Table 1:** Critical temperatures and relative errors for the lattice dimensions 3≤d≤7. Results of mean field theory are also shown.

	$\mathrm{Critical}\;\mathrm{Temperature}\;\beta_{c}$				
	*d* = 3	*d* = 4	*d* = 5	*d* = 6	*d* = 7
Best numerical results [9,21,23]	0.22166	0.14970	0.11392	0.09230	0.07771
Account for third moment, Equation (40), k=−0.8	0.22155	0.14894	0.11372	0.09227	0.07772
Gaussian approximation (18)	0.21132	0.14645	0.11270	0.09175	0.07742
Account for third moment, Equation (40), k=1	0.20196	0.14366	0.11151	0.09113	0.07706
Mean field theory	0.16667	0.12500	0.10000	0.08333	0.07143
	**Relative error** Err				
Account for third moment, Equation (40), k=−0.8	0.02%	0.25%	0.09%	0.02%	0.01%
Gaussian approximation (18)	2.39%	1.10%	0.54%	0.30%	0.18%
Account for third moment, Equation (40), k=1	4.65%	2.06%	1.07%	0.64%	0.42%
Mean field theory	14.16%	8.99%	6.50%	5.10%	4.21%

**Table 2:** Values measured at $\beta =\beta_{\max}$.

$J_{NNN}$	$J_{NNNN}$	q	$\beta_{\max}$	$\sigma_{\max}^{2}$	|〈E〉|	$\langle E^{2}\rangle$	$\langle E^{4}\rangle$	|〈m〉|	$\langle m^{2}\rangle$	$\langle m^{4}\rangle$	$Q_{\max}$
0.0000	0	6.00	0.22208	71.02	1.0247	1.0502	1.1041	0.06448	0.04742	0.00258	0.6173
0.0042	0	6.10	0.21978	74.45	1.0270	1.0550	1.1143	0.10751	0.04862	0.00271	0.6173
0.0410	0	7.00	0.20058	92.80	1.0423	1.0866	1.1823	0.11810	0.05241	0.00310	0.6243
0.0811	0	8.00	0.18328	107.06	1.0517	1.1065	1.2260	0.14503	0.04908	0.00273	0.6223
0.1213	0	9.00	0.16888	132.16	1.0623	1.1290	1.2770	0.08342	0.04356	0.00223	0.6084
0.1623	0	10.00	0.15648	129.91	1.0834	1.1743	1.3813	0.17143	0.04511	0.00229	0.6252
0.2048	0	11.00	0.14548	154.83	1.1020	1.2151	1.4792	0.15042	0.04237	0.00206	0.6184
0.2500	0	12.00	0.13548	189.87	1.1300	1.2776	1.6360	0.10335	0.04287	0.00212	0.6154
0.2991	0	13.00	0.12608	199.76	1.1546	1.3339	1.7833	0.01969	0.03976	0.00183	0.6145
0.3543	0	14.00	0.11709	243.49	1.1972	1.4342	2.0623	0.07870	0.04215	0.00203	0.6194
0.4189	0	15.00	0.10799	287.88	1.2179	1.4844	2.2101	0.06442	0.03106	0.00123	0.5765
0.5000	0	16.00	0.09869	325.40	1.3223	1.7497	3.0701	0.08284	0.04788	0.00249	0.6373
0.6169	0	17.00	0.08769	426.66	1.4011	1.9647	3.8730	0.03163	0.03256	0.00142	0.5536
0.7106	0	17.50	0.08059	482.31	1.4871	2.2134	4.9152	0.12453	0.04236	0.00200	0.6290
0.8566	0	17.90	0.07149	586.79	1.5548	2.4198	5.8771	0.02487	0.02444	0.00080	0.5519
0.8958	0	17.95	0.06949	600.78	1.6359	2.6783	7.1979	0.03046	0.03645	0.00151	0.6216
0.9324	0	17.98	0.06769	655.33	1.6793	2.8225	7.9946	0.03795	0.03277	0.00129	0.5992
1.0000	0.0000	18.00	0.06448	736.77	1.6900	2.8587	8.2047	0.01656	0.02573	0.00087	0.5643
1	0.0063	18.10	0.06429	768.80	1.6916	2.8644	8.2385	0.06596	0.03199	0.00123	0.5993
1	0.0637	19.00	0.06248	770.88	1.7294	2.9938	8.9982	0.07366	0.03418	0.00136	0.6133
1	0.1307	20.00	0.06049	848.33	1.7475	3.0569	9.3844	0.03053	0.03480	0.00140	0.6135
1	0.2023	21.00	0.05848	904.70	1.7442	3.0458	9.3187	0.08452	0.03413	0.00135	0.6132
1	0.2806	22.00	0.05649	926.04	1.7881	3.2008	10.2901	0.18149	0.03500	0.00150	0.5918
1	0.3693	23.00	0.05439	989.95	1.8190	3.3125	11.0228	0.08570	0.04307	0.00201	0.6390
1	0.4755	24.00	0.05199	1060.91	1.7446	3.0478	9.3385	0.00448	0.02501	0.00080	0.5762
1	0.6177	25.00	0.04919	1157.27	1.7578	3.0944	9.6306	0.01029	0.02170	0.00063	0.5537
1	0.7225	25.50	0.04739	1324.75	1.8598	3.4641	12.0698	0.03932	0.03389	0.00131	0.6203
1	0.8707	25.90	0.04499	1418.32	1.8758	3.5239	12.4942	0.09043	0.02928	0.00101	0.6080
1	0.9076	25.95	0.04449	1414.33	1.9694	3.8839	15.1680	0.20027	0.04111	0.00182	0.6402
1	0.9410	25.98	0.04399	1455.97	1.9562	3.8324	14.7722	0.15120	0.03737	0.00154	0.6334
1	1.0000	26.00	0.04309	1505.02	1.8808	3.5433	12.6366	0.01037	0.02359	0.00071	0.5765

## Data Availability

Not applicable.

## Appendix A. Calculation of the First Moments of Energy Distribution

Previously, we described in detail how to calculate the moments of the distribution of the energy states belonging to the vicinity $\Omega_{m}$ [26,27,28]. In these papers, we obtained the exact expressions for the first two moments of the energy distribution that are the mean $E_{m}$ and the variance $\sigma_{m}^{2}$. For a finite system and a general connection matrix $J={\left(J_{ij}\right)}_{i,j=1}^{N}$, we presented these expressions in finitely combinatorial forms. In the case of the Ising connection matrix for a d-dimensional hypercube, we, in addition, found $E_{m}$ and $\sigma_{m}^{2}$ in an asymptotic limit when the number of spins N tends to infinity. Next, we show how to obtain the combinatorial and the asymptotic formulas for the third moment $\mu_{3}$.

### Appendix A.1. Notations

In what follows, for the sake of simplicity when averaging over the *m*-vicinities $\Omega_{m}$, we sometimes will omit the subscript *m*. For example, if a state $s=(s_{1},s_{2},\ldots ,s_{N})$ belongs to an *m*-vicinity $\Omega_{m}$ and E(s) is the energy of the state s, we will write the first three moments as

$$\langle E\rangle =N_{m}^{-1}\sum_{s\in \Omega_{m}}E(s),\;\langle E^{2}\rangle =N_{m}^{-1}\sum_{s\in \Omega_{m}}E^{2}(s),\;\mathrm{and}\;\langle E^{3}\rangle =N_{m}^{-1}\sum_{s\in \Omega_{m}}E^{3}(s),\;\mathrm{where}\;N_{m}=\left(\begin{array}{c} N\\ n \end{array}\right).$$

We are interested in calculating the second and third central moments—the variance $\sigma^{2}$ and the semi-invariant $\mu_{3}$:

$$\mu_{3}=\langle E^{3}\rangle -3\sigma^{2}\langle E\rangle -{\langle E\rangle }^{3},\;\mathrm{and}\;\sigma^{2}=\langle E^{2}\rangle -{\langle E\rangle }^{2}.$$

Usually, the energy $E(s)=a\cdot \sum_{i,j=1}^{N}J_{ij}s_{i}s_{j}$ of the state s includes a normalization coefficient a. In what follows, we for simplicity omit this coefficient and write the energy as

(A1) $$E(s)=sJs^{+}\equiv \sum_{i,j=1}^{N}J_{ij}s_{i}s_{j}.$$

After obtaining the final expressions for the moments, we have to multiply $\langle E\rangle ,\;\sigma^{2}$, and $\mu_{3}$ by a, $a^{2}$, and $a^{3}$, respectively.

### Appendix A.2. Connection Matrix of General Form

Suppose that the connection matrix has a general form and the configuration $s_{0}=(s_{01},s_{02},\ldots ,s_{0N})$ is the ground state whose energy is $E_{0}=s_{0}Js_{0}^{+}$.

To calculate the energy moments $\langle E^{r}\rangle$, r=1,2,…, it is necessary to present $E^{r}$ as a sum of terms which contain only the products of spin variables with non-repeating indices. For example, we can write the expressions for E and $E^{2}$ as

$$E=\sum_{i=1}^{N}\sum_{j=1}^{N}J_{ij}s_{i}s_{j}\chi_{ij}$$

$$E^{2}=2\mathrm{Tr}J^{2}+4\sum_{i,j=1}^{N}{\left(J^{2}\right)}_{ij}s_{i}s_{j}\chi_{ij}+\sum_{i,j,k,r=1}^{N}J_{ij}J_{kr}s_{i}s_{j}s_{k}s_{r}\chi_{ijkr},$$

where $\chi_{ij}$ and $\chi_{ijkr}$ are tensors, whose components are nonzero and equal to one only if they do not contain the repeating indices.

In the *m*-vicinity, we can present the variables $s_{i}$ as $s_{i}=s_{0i}p_{i}$, where $p_{i}$ takes the value $p_{i}=-1$ with the probability of n/N and it takes the value $p_{i}=1$ with the probability (N−n)/N. (We remind that n=N(1−m)/2.) This allows us to easily perform the averaging over the *m*-vicinities. For example:

(A2) $$\langle E\rangle =N_{m}^{-1}\sum_{\Omega_{m}}\sum_{i=1}^{N}\sum_{j=1}^{N}J_{ij}s_{0i}s_{0j}p_{i}p_{j}\chi_{ij}=\sum_{i=1}^{N}\sum_{j=1}^{N}J_{ij}s_{0i}s_{0j}\cdot N_{m}^{-1}\sum_{\Omega_{m}}p_{i}p_{j}\chi_{ij}=\;E_{0}K_{2}.$$

When writing Equation (A2), we used easily verifiable formulas $N_{m}^{-1}\sum_{\Omega_{m}}p_{i}p_{j}\chi_{ij}=K_{2}\chi_{ij}$, $N_{m}^{-1}\sum_{\Omega_{m}}p_{i}p_{j}p_{k}p_{r}\chi_{ijkr}=K_{4}\chi_{ijkr}$, and $N_{m}^{-1}\sum_{\Omega_{m}}p_{i}p_{j}p_{k}p_{r}p_{m}p_{n}\chi_{ijkrmn}=K_{6}\chi_{ijkrmn}$:

(A3) $$\begin{array}{l} K_{2}=\frac{{\left(N-2n\right)}^{2}-N}{N(N-1)},\;\;\;\;\;\;\;\;\;\;\;\;\\ K_{4}=\frac{{\left(N-2n\right)}^{4}-2{\left(N-2n\right)}^{2}(3N-4)+3N(N-2)}{N(N-1)(N-2)(N-3)},\\ K_{6}=1-\frac{4(N-n)\cdot n\cdot B}{N(N-1)(N-2)(N-3)(N-4)(N-5)}.\; \end{array}$$

In Equation (A3), the constant B in the numerator of the last expression is equal to

$$\begin{array}{ll} B= & {3(N-2n)}^{4}+6{\left(N-2n\right)}^{3}\cdot (2n-5)+{\left(N-2n\right)}^{2}\cdot (28n^{2}-120n+125)+\\ & +(N-2n)(32n^{3}-180n^{2}+340n-210)+4(n-1)(n-2)(4n^{2}-18n+23). \end{array}$$

The same as when calculating <E> in Equation (A2), we can obtain the second and the third energy moments and show that the averaging over $\Omega_{m}$ leads to the following equalities:

(A4) $$\begin{array}{l} E_{m}=K_{2}E_{0},\;\;\;\;\;\;\;\;\;\;\;\;\;\;\;\;\;\;\;\;\;\;\;\;\;\;\;\;\;\;\;\\ \sigma_{m}^{2}=2(1-2K_{2}+K_{4})\cdot \mathrm{Tr}\;J^{2}+4(K_{2}-K_{4})\cdot \left(s_{0}J^{2}s_{0}^{+}\right)-(K_{2}^{2}-K_{4})E_{0}^{2},\;\;\;\;\;\;\;\;\;\\ \mu_{3}(m)=8\left(1-3K_{2}+3K_{4}-K_{6}\right)\cdot \mathrm{Tr}\;J^{3}-16(K_{4}-K_{6})\cdot \sum_{i=1}^{N}s_{0i}h_{0i}^{3}-\;\;\;\;\;\;\;\;\;\;\;\\ \;-12E_{0}\left(s_{0}J^{2}s_{0}^{+}\right)(K_{2}^{2}-K_{4}-K_{2}K_{4}+K_{6})+6E_{0}\mathrm{Tr}\;J^{2}\left[K_{6}-K_{2}K_{4}+2(K_{2}^{2}-K_{4})\right]+\\ +E_{0}^{3}\left(K_{6}-3K_{2}K_{4}+2K_{2}^{3}\right)+8\left(K_{2}-2K_{4}+K_{6}\right)\left[2\left(s_{0}J^{\left(3\right)}s_{0}^{+}\right)-6\sum_{i=1}^{N}s_{0i}h_{0i}\cdot {\left(J^{2}\right)}_{ii}+3\left(s_{0}J^{3}s_{0}^{+}\right)\right], \end{array}$$

where $h_{0i}=\sum_{j=1}^{N}J_{ij}s_{0j}$ is a local field acting on the *i*-th spin belonging to state $s_{0}$. The matrix $J^{\left(3\right)}$ in the expression for the third moment is $J^{\left(3\right)}=\left(J_{ij}^{3}\right)$.

The expressions (A3) and (A4) provide us with the most general formulas for the first, second, and third moments of the distribution of energies of the states from the *m*-vicinity $\Omega_{m}$ in terms of the elements of the connection matrix J. (If it is necessary to use the normalized expression for the energy E(s), see the note after Equation (A1).)

### Appendix A.3. Ising Model on Hypercube

In this subsection, we analyze the Ising model with the short-range interaction, when only the nearest spins interact, and other elements of the connection matrix are equal to zero. Let the nonzero matrix elements be equal to a constant J. We factor out this constant in all our calculations. Then, there are q=2d ones in each row of the matrix J, all the other matrix elements are equal to zero, and the ground state is a configuration $s_{0}=(1,1,1,\ldots ,1)\in R^{N}$.

It is easy to see that the following equations

$$\begin{array}{l} E_{0}=qN,\;\;\mathrm{Tr}\;J^{2}=qN,\;\;s_{0}J^{2}s_{0}^{+}=q^{2}N,\;\;\mathrm{Tr}\;J^{3}=0,\\ \sum_{i=1}^{N}s_{0i}h_{0i}\cdot {\left(J^{2}\right)}_{ii}=q^{2}N,\;s_{0}J^{3}s_{0}^{+}=q^{3}N,\;\sum_{i=1}^{N}s_{0i}h_{0i}^{3}=q^{3}N. \end{array}$$

hold. Then the expressions (A4) take the form

$$E_{m}=qN\cdot K_{2}$$

(A5) $$\sigma_{m}^{2}=qN\cdot \left\{qN\cdot (K_{4}-K_{2}^{2})+2\left[1-2K_{2}+K_{4}+4q(K_{2}-K_{4})\right]\right\}$$

$$\begin{array}{l} \mu_{3}(m)={\left(qN\right)}^{3}\left(K_{6}-3K_{2}K_{4}+2K_{2}^{3}\right)+\\ \;\;\;+6\;{\left(qN\right)}^{2}\left[K_{2}^{2}-K_{4}+(1-2q)\left(K_{2}^{2}-K_{4}+K_{6}-K_{2}K_{4}\right)\right]+\\ \;\;\;+8\;(qN)\left[\left(K_{2}-K_{4}\right)\left(2-6q+3q^{2}\right)+\left(K_{6}-K_{4}\right)\left(2-6q+5q^{2}\right)\right]. \end{array}$$

To obtain asymptotic expressions for the moments (N→∞), at first it is necessary to obtain the asymptotic expressions for the coefficients $K_{2}$, $K_{4}$, and $K_{6}$, expanding them with respect to the small parameter δ=1/N:

$$\begin{array}{l} K_{2}\approx m^{2}-\delta \cdot (1-m^{2})-\delta^{2}(1-m^{2}),\\ K_{4}\approx m^{4}-\delta \cdot 6m^{2}(1-m^{2})+\delta^{2}(1-m^{2})(3-25m^{2}),\\ K_{6}\approx m^{6}-\delta \cdot 15m^{4}(1-m^{2})+\delta^{2}5m^{2}(1-m^{2})(9-28m^{2}). \end{array}$$

here, m=(N−2n)/N∈ [−1,+1] is the magnetization of the state $s\in \Omega_{m}$.

Next, substituting the obtained expressions into Equation (A5) and leaving the leading terms, we obtain the asymptotic expressions for the first three moments

(A6) $$\begin{array}{l} E_{m}=N\cdot qm^{2},\\ \sigma_{m}^{2}=N\cdot 2q{\left(1-m^{2}\right)}^{2},\\ \mu_{3}(m)=N\cdot 16qm^{2}{\left(1-m^{2}\right)}^{2}. \end{array}$$

The final answer we obtain after multiplying $E_{m}$, $\sigma_{m}^{2}$, and $\mu_{3}(m)$ by a, $a^{2}$, and $a^{3}$, respectively; here, a=−J/2N.

### Appendix A.4. Asymptotic Form of Coefficient $\kappa_{3}$

Starting from Equation (8), we use the series expansion of the unknown function φ(m,E) in the variable $\varepsilon =(E-E_{m})/\sigma_{m}$:

(A7) $$\varphi =\frac{1}{2}\varepsilon^{2}+\frac{1}{3!}\kappa_{3}\varepsilon^{3}+\frac{1}{4!}\kappa_{4}\varepsilon^{4}+\ldots$$

Since we do not know the function φ(m,E) itself, we also do not know the series expansion coefficients $\kappa_{r}$, where r=3,4,… However, we do know the first three moments of the energy distribution (A5) and this allows us to define the coefficient $\kappa_{3}$ of the expansion (A7). Indeed, Equations (A5) and (A6) define the general form of the third moment:

(A8) $$\mu_{3}(m)=\int {\left(E-E_{m}\right)}^{3}\exp\left[-N\cdot \varphi (m,E)\right]dE.$$

We restrict ourselves with the first two terms in the right-hand side of Equation (A7) and substitute the expressions (A5)–(A7) into the integral in the right-hand side of Equation (A8). Then

(A9) $$\mu_{3}(m)=\sigma_{m}^{4}\int \exp\left[-N\cdot \left(\frac{1}{2}\varepsilon^{2}+\frac{1}{3!}\kappa_{3}\varepsilon^{3}\right)\right]\varepsilon^{3}d\varepsilon$$

It is easy to see that the main contribution to the integral (A9) comes from the region $\varepsilon \sim \pm N^{-1/2}$, where the cubic term of the exponent of the integrand is negligible small $\left(N\varepsilon^{3}\sim N^{-1/2}\right)$. Then expanding the exponent, we have

(A10) $$\mu_{3}(m)=\sigma_{m}^{4}\int \exp\left(-\frac{1}{2}N\varepsilon^{2})\cdot (1-\frac{1}{3!}N\kappa_{3}\varepsilon^{3}\right)]\varepsilon^{3}d\varepsilon$$

After simple calculations, equating expression (A10) and the third of the expressions (A6), we obtain, with an accuracy up to the terms of the order of $O\left(N^{-1}\right)$,

(A11) $$\kappa_{3}=-\frac{\mu_{3}(m)}{\sigma_{m}^{3}}=\frac{2qm^{2}}{\sigma_{0}^{3}\left(1-m^{2}\right)}.$$

In the same way, it is also possible to calculate the coefficients of the higher order. However, even the expression for $\kappa_{4}$ is so cumbersome that we do not present it here.
